# Coronary Artery Bypass Grafting in a Patient With Severe Factor VII Deficiency

**DOI:** 10.7759/cureus.37815

**Published:** 2023-04-19

**Authors:** Yagya Ahlawat, Rathnamitreyee Vegunta, Nirali Sanghavi, John Nelson

**Affiliations:** 1 Internal Medicine, Westchester Medical Center, Valhalla, USA; 2 Hematology and Oncology, Westchester Medical Center, Valhalla, USA

**Keywords:** cabg surgery, bleeding risk, elevated inr, clotting disorder, coronary artery bypass grafting(cabg), factor 7 deficiency

## Abstract

Factor VII deficiency is a rare bleeding disorder. Clinical presentation is highly variable and can range from mild symptoms like mucosal bleeding to life-threatening hemorrhages in early infancy. Some people remain asymptomatic and are only diagnosed incidentally on laboratory tests. Given the low incidence in the population and variable phenotypes, there are no official guidelines on the management of such patients perioperatively to minimize bleeding risk. We present a case of a man with inherited severe factor VII deficiency who underwent successful coronary artery bypass grafting.

## Introduction

Factor VII is a vitamin-K-dependent clotting protein that participates in the extrinsic pathway of the coagulation cascade. Its inherited deficiency is a rare autosomal recessive disorder affecting about one in 500,000 people [1]. It is due to a mutation within the F7 gene present on chromosome 13 [2]. In laboratory tests, there is an isolated prolongation of prothrombin time, with normal partial thromboplastin time. Interestingly, the risk of bleeding in these patients does not seem to correlate with factor VII activity in plasma [1]. Given the rarity of this disease and its variable presentation, optimal perioperative management to minimize bleeding risk is unclear. Laboratory measures like prothrombin time and activity of factor VII help in diagnosing the deficiency, but their absolute values do not predict the risk of bleeding for these patients. This makes optimization prior to surgery challenging. So far there are only a few reported cases of individuals with factor VII deficiency who underwent coronary artery bypass grafting.

## Case presentation

A 64-year-old man was admitted to the hospital to undergo left heart catheterization (LHC) for stable angina. He was recently incidentally diagnosed with an isolated inherited factor VII deficiency. His past medical history was significant for type two diabetes, hypertension, and benign prostatic hyperplasia. He had no history of excessive bruising or bleeding. His family history was negative for any bleeding disorder. Laboratory test results were significant for an elevated prothrombin time (32.2 seconds), markedly elevated international normalized ratio (INR) (5.68), normal partial thromboplastin time (32.2 seconds), and factor VII activity <0.5%. On next-generation sequencing, he was found to have homozygous pathogenic variant F7 (DNA change c.1109G>T; amino acid change p.Cys370Phe).

Hematology was consulted for the optimization of the INR. He received two units of fresh frozen plasma (FFP) prior to LHC. He underwent successful LHC which showed severe multivessel coronary artery disease, for which he was subsequently planned for coronary artery bypass graft. Elevated INR posed a dilemma for perioperative management. INR was noted to be 6.4 on the day of the surgery. He received two units of FFP before the start of the procedure followed by continuous FFP similar to a drip concept to provide a small amount of continuous factor VII (by running one unit of FFP over four hours for 24 hours). Per blood bank policy at our institution, once transfusion of a bag of blood product is initiated, it must be completed within four hours, which is why this was the slowest rate at which we could administer FFP. This was done to minimize the volume of plasma transfused, as there was a risk of transfusion-associated circulatory overload.

There was no excessive intraoperative bleeding. INR ranged from 2.19 to 2.42 on the day of the procedure. Post-operatively, his INR rose to his baseline, which is around 6. He was deemed safe to be started on aspirin. Later in his hospital course, he had a chest tube placed while his INR ranged from 7 to 8 without giving any additional blood products. He had no clinical evidence of bleeding and tolerated all procedures well.

## Discussion

Multiple studies have demonstrated that plasma activity levels of factor VII cannot be used to predict the risk of bleeding as the clinical presentation is very heterogeneous. Severe phenotypes can present with life-threatening bleeding within the first six months of life (for example, intracranial and gastrointestinal hemorrhages), and milder presentation can range from epistaxis to traumatic hemarthrosis [1]. Personal history of bleeding is a more reliable indicator of the risk of severe bleeding during surgery. There have been cases reported with factor VII activity of less than 1% and no personal history of bleeding similar to our patient who successfully underwent minor surgical procedures without the need for replacement therapy [3,4].

At present, there are various therapeutic modalities available for inherited factor VII deficiency, which include recombinant activated factor VII, plasma-derived factor VII, FFP, and prothrombin complex concentrates. The choice of treatment depends upon various factors including the severity of the disease, other co-morbidities, availability of products, and economic factors [5].

Rosenthal et al. reported successful coronary artery bypass grafting in a case of severe congenital factor VII deficiency, which was managed by giving factor VII concentrate perioperatively [6]. As in our case, FFP can be given if factor VII is not readily available, but there is a risk of fluid overload with multiple infusions [5].

## Conclusions

Inherited factor VII deficiency is a rare cause of bleeding. Such patients have isolated elevation of prothrombin time (PT)/INR on the coagulation profile. Perioperative optimization for major cardiothoracic surgery in these patients is challenging as laboratory abnormalities do not accurately predict bleeding risk. Therefore, a multidisciplinary approach is necessary for effective management. In our experience and based on prior case reports of similar patients undergoing coronary artery bypass grafting, personal history of bleeding is a more reliable indicator. One of the ways of dealing with factor VII deficiency in a clinically asymptomatic individual as in our case is by giving a slow continuous infusion of FFP intraoperatively. It is less expensive and more readily available compared to factor VII concentrate. Further studies are needed to see if there is any benefit of targeting a specific INR prior to surgery in such cases.
